# Sagittal spinal alignment and ambulation

**DOI:** 10.1186/1748-7161-10-S1-O26

**Published:** 2015-01-19

**Authors:** Keiji Ishii, Kazuhiro Hasegawa, Haruka Shimoda, Takao Honma

**Affiliations:** 1 Niigata Spine Surgery Center, Niigata, Japan

## 

A relationship between spinal deformity and ambulation is yet to be clarified. We hypothesized that undesirable spino-pelvic alignment affects walking ability. The purpose of present study is to clarify an influence of spino-pelvic alignments on walking ability. Consecutive patients who visited our institution between October 2011 to September 2012 were retrospectively surveyed. The cases of neuromuscular or cerebrovascular disorder and spinal fusion or arthroplasty were excluded. Finally, a total of the 176 patients matched the inclusion criteria and were enrolled in this study. They were divided into 2 groups. Group G consists of 126 patients (male:53 female:71, average age:75.3) walking without supportive tool. Group I consists of 51 patients (male:15 female:36, average age:79.4) walking with supportive tool. Their diagnosis, past history, bone mineral density and spino-pelvic alignments (Coronal Cobb angle, C7 coronal offset, C2-7 angle, thoracic kyphosis, Lumbar Lordosis, Sacral Slope, Pelvic tilt, Pelvic Incidence, Overhang, T9SPI, T1SPI and SVA) were analyzed and compared by Fisher's exact test and Wilcoxon-rank sum test. AP value of less than 0.05 was considered statistically significant. No significant difference was observed between their frequency of the diagnosis, past history, the value of bone mineral density, coronal alignments, C2-7 angle, thoracic kyphosis, pelvic incidence and T9SPI. There were, however, significant difference between their Sacral Slope (group G: 24.5° vs Group I: 19.9°), Pelvic tilt (group G: 26.2° vs Group I: 32.3°) and Overhang (group G: 46.2mm vs Group I: 57.1mm) (P<0.05). Further, there were significant difference between their Lumbar Lordosis (group G: 32.6° vs Group I: 17.0°), T1SPI (group G: 2.1° vs Group I: -3.2°) and S VA (group G: 49.0mm vs Group I: 109mm), indicating the reduction of lordosis and stooped posture (P<0.01). The results in present study support the previous reports about the relation between spino-pelvic alignments and quality of life and also correspond with the hypothesis that the change of pelvic inclination compensates the decrease of lordosis and further degeneration leads to anterior trunk inclination, requiring a supportive tool during walking, suggesting that the patients would decline activity in daily living and decline their general health.

